# Technology-driven diabetes care: innovation without equity?

**DOI:** 10.3389/fdgth.2026.1783456

**Published:** 2026-03-20

**Authors:** Izere Salomon, Shema Sam

**Affiliations:** 1 Department of General Medicine and Surgery, College of Medicine and Health Sciences, University of Rwanda, Kigali, Rwanda; 2 University Teaching Hospital of Butare (CHUB), Butare, Rwanda

**Keywords:** artificial intelligence in healthcare, continuous glucose monitoring (CGM), diabetes care, digital divide, digital health (DH), health equity, low- and middle-income countries (LMICs), type 1 and type 2 diabetes

With over 500 million people affected globally, diabetes has become one of the most urgent and inequitable chronic conditions of our time (1), and innovative digital tools, from continuous glucose monitors (CGMs) and smart insulin pumps to mobile apps and AI-driven screening, promise more precise, personalized management (2). Research has consistently shown that these technologies can markedly improve glycemic control and quality of life (3). For example, smartphone-based educational programs guided by AI have led to significantly better HbA_1_c reduction than standard care (4), and CGM systems enable real-time monitoring that supports timely insulin adjustment (2).

Diabetes mellitus type 1, commonly known as type 1 diabetes (T1D), is an autoimmune disease that occurs when the body's immune system destroys insulin-producing β-cells in the pancreas, leading to little or no endogenous insulin production (5). People with T1D (often diagnosed in childhood) require lifelong insulin therapy to remain alive (5). In contrast, diabetes mellitus type 2, also commonly known as type 2 diabetes (T2D), is a form of diabetes mellitus characterized by insulin resistance and relative insulin deficiency (5). Most patients with T2D are initially managed with lifestyle changes and oral medications, with insulin reserved for later stages or in special situations. T2D is far more common than T1D, accounting for approximately 90%–95% of all diabetes cases (5), and is strongly associated with obesity and aging. These distinctions affect technology use; advanced devices such as insulin pumps and automated “artificial pancreas” systems have been developed primarily for insulin-dependent patients, whereas T2D care more often uses glucose meters, CGMs, telemedicine, and mobile apps to support lifestyle and medication adherence. Notably, digital interventions have been shown to improve glycemic control in both T1 and T2Ds (6), but access and uptake have not been equal in the largely non-insulin-dependent T2 population.

In theory, these advancements can reduce complications and costs. However, in practice, the adoption of digital diabetes technologies is significantly uneven, with poorer countries lagging far behind wealthier nations in the use of pumps, continuous glucose monitors (CGMs), telemedicine, and AI tools. This disparity raises urgent questions regarding equity.

## A global divide in access and outcomes

These patterns reflect the well-known equity principles. In Tudor Hart's classic “inverse care law,” new health innovations tend to reach affluent populations first, leaving high-need groups behind (7). In diabetes, this effect is amplified by a persistent digital divide; low-income and rural patients face financial, educational, and connectivity barriers to using CGMs, pumps, or telehealth, whereas wealthier patients rapidly adopt these tools (7, 8). Moreover, data-driven tools risk algorithmic bias; AI models trained on data from well-resourced settings may misdiagnose or undertreat patients from marginalized communities (9). This dynamic is compounded by regulatory and contextual inequities; for example, technologies developed in high-income countries are often implemented in low-resource areas without local adaptation, a form of “digital colonialism” (9). Therefore, without paying attention to these systemic factors (inverse care law, digital access, biased algorithms, and uneven regulation), new diabetes technologies may inadvertently widen global disparities instead of closing them (7, 9).

Increasing evidence highlights this disparity. A 2025 JAMA Network study, which focused on pediatric diabetes care through the SWEET initiative, found that “rapid advancement in diabetes technologies has resulted in global disparities in terms of access” (3). High-income countries (HICs) now reimburse advanced systems, whereas low- and middle-income countries (LMICs) often limit access to basic glucometers or have no devices at all. Indeed, the WHO reports that “on a global level, 1 out of every 2 people needing insulin for T2D does not get this essential medicine” (10) and that insulin consumption in LMICs does not keep pace with the epidemic. In Europe alone, one analysis found that reimbursement policies for pumps and CGMs were highly heterogeneous, even among affluent countries (3), a microcosm of wider inequities. Globally, centers with full technology access achieve far lower HbA₁c levels than those with limited resources (3). Such data clarify that unequal access to technology translates into unequal health outcomes; countries still struggle with the basic issue of insulin availability, much less “closed-loop” artificial pancreas therapy (3).

LMICs face significant challenges in terms of diabetes care. More than 80% of diabetes cases are now found in these regions (11), yet they often lack adequate screening and treatment infrastructure. For instance, diabetic eye care, where smartphone fundus cameras and AI grading could make a significant difference, is largely unavailable on a large scale; only one of the 50 LMICs has a national diabetic retinopathy screening program (11). A recent review of screening in South Asia and Africa found that community-based telemedicine and AI-enabled imaging improved coverage and cost-effectiveness, yet are still pilot efforts in resource-poor settings (11). Mobile health initiatives show promise (12), but such successes remain isolated and often depend on external funding or NGOs.

## Drivers of the digital divide

Several factors have contributed to the digital divide (Table 1). Cost was one of the most significant factors. Advanced devices and software can be quite expensive; for example, a CGM sensor can cost hundreds of dollars in just a few weeks of use. Additionally, insulin pumps and AI-based diagnostic systems require expensive hardware and maintenance. Thus, out-of-pocket payments remain the norm in many countries (3). Additionally, reliable Internet and cellular connectivity, which many applications and telehealth tools require, are not widely available. In the US, both poor urban and rural populations have limited broadband access, and Internet connectivity is a significant social determinant of health for people with diabetes (8). In sub-Saharan Africa and South Asia, where smartphone ownership and data plans lag, simple mHealth programmes struggle to engage users.

**Table 1:** Barriers and solutions for equitable diabetes technology.

Barrier	Description	Proposed solutions
Cost and affordability	High price of CGMs, pumps, and mobile platforms	Subsidies, pooled procurement, tiered pricing
Digital exclusion	Poor internet, low smartphone penetration, tech literacy gaps	Offline-capable tools, community health worker (CHW) training, and public broadband access
Clinical capacity gaps	Lack of trained staff, overburdened systems	Task-shifting, e-learning modules, digital decision aids
Regulatory barriers	No pathways for software approval, lack of interoperability standards	WHO-aligned standards, local regulatory capacity building
Cultural/contextual mismatch	Devices not adapted to local languages, beliefs, or care settings	Co-creation with communities, participatory design
Algorithmic bias	AI trained on non-representative populations	Federated learning, diverse datasets, fairness auditing

Beyond economics, health system readiness and regulations pose barriers. Many LMIC clinics lack trained staff to use or interpret digital data; as one survey noted, even adequate clinic staffing and multidisciplinary teams contribute greatly to outcomes (3). Physicians in underresourced health systems have little time to introduce or monitor new devices. Regulatory capacity is also limited; few LMIC regulators have clear pathways for approving novel medical software or ensuring interoperability; therefore, companies have little incentive to market devices there. Meanwhile, cultural and informational gaps, such as language, health literacy, and trust in technology, can dampen demand. If apps and interfaces are not tailored to the local languages and contexts, patients may ignore them. As one expert warns, devices designed for one population often underperform in another, a form of “data colonialism.” For instance, an AI algorithm trained on white patients’ retinal images showed reduced sensitivity in darker-skinned populations (4). Similarly, wearables and smartphone-driven models risk bias; people without smartphones or with different lifestyles may be sidelined (4).

## Consequences of unequal access

The impact of the digital divide on human life is significant. Without access to modern tools, low-income patients experience higher rates of complications and mortalities. In the Americas, the prevalence of diabetes has tripled, and it now ranks as the sixth leading cause of death (10). Technical models predict that suboptimal monitoring accelerates blindness, kidney failure, and amputations (10). Indeed, the WHO explicitly notes that insulin, the simplest diabetes “technology,” is vital to prevent kidney and eye damage in millions of T2D patients (10), yet remains unaffordable for many.

Insulin is a life-saving therapy for T2D patients with marked insulin deficiency, for example, in late-stage disease or pregnancy, but its risks and benefits must be carefully weighed. Observational studies have reported higher rates of cardiovascular events and mortality in type 2 cohorts treated with insulin than in those treated with other regimens (13). However, large randomized trials (such as long-term trials of basal insulin) generally found no increase in cardiovascular or all-cause mortality (14). Meta-analyses have not shown a consistent harmful effect of insulin on these outcomes (14). Taken together, the evidence suggests that insulin itself is not intrinsically dangerous, but its use in heterogeneous patient populations can reflect the underlying illness severity. Thus, initiation of insulin in T2D should be individualized, and clinicians should consider each patient's comorbidities, risks of hypoglycemia, and glucose targets, and should monitor closely after starting insulin (13, 14).

Uncontrolled diabetes also strains health budgets; worldwide, diabetes care already costs nearly $1 trillion annually (2) and unchecked diseases will only expand that. Thus, technological inequity worsens health disparities; innovations meant to help may arrive too late for the most disadvantaged, further entrenching global health issues (3, 10).

## Bridging the gap: inclusive innovation and partnerships

Addressing the equity gap requires bold action and collaboration (Table 1). A crucial first step is inclusive design, which involves co-creating technologies along with communities and local health systems. The Diabetes Compass Initiative of the World Diabetes Foundation offers this model. Malawi, Tanzania, and Sri Lanka worked with ministries and local providers to co-develop digital training and screening tools tailored to each country's needs (15). Offline mobile apps were created as global reference solutions but were fully customizable for local guidelines (15), empowering clinicians with accessible e-learning and decision aids. Such approaches blending global expertise with local ownership leverage public–private partnerships to subsidize costs and build capacity. Similarly, pooled procurement mechanisms such as PAHO's Strategic Fund can drive down prices for insulin and supplies, whereas grant funding can offset the cost of early digital deployments.

Bridging this equity gap requires context-sensitive solutions. The WHO and other experts stress that health technologies must be appropriate, affordable, and safe for low-resource settings (16). Practical examples include solar-powered refrigerators for insulin, low-cost glucometers, and SMS-based glucose-monitoring systems designed with community input. Crucially, these interventions should be co-created with patients, local clinicians, and communities. For instance, the Diabetes Compass initiative engages national diabetes associations as *co-creators* and “incubators” of digital solutions, ensuring that each tool addresses an identified local need (17). Participatory design and social innovation, involving end users at every stage, help tailor devices and programs to cultural norms and infrastructure. In this way, technology serves as an enabler of care rather than an inaccessible novelty. By building solutions from the ground up, programs can leverage high-tech tools in a way that is truly useful and sustainable in each setting (16, 17).

Standard setting is also critical. Global bodies are moving in this direction; the WHO's Global Strategy on Digital Health explicitly calls for innovations to support “equitable and universal access” to care (18). International guidelines on AI fairness and data sharing should be rapidly developed and adopted, ensuring that new diabetes tools meet standards for validity across diverse populations. Technical solutions, such as federated learning, where AI models learn from data across many countries without sharing raw data, can help create more robust and unbiased algorithms (4). Interoperability standards would allow low-cost devices to be plugged into electronic records and national registries.

Ultimately, we must nurture capacity building in LMICs, train health workers in digital skills, improve broadband connectivity, and educate patients on technology. Education campaigns can increase trust and uptake, just as training village health teams has begun to do in Uganda (12). Donors and governments should invest in infrastructure such as electricity, the Internet, and others as health priorities, just as they do for roads or water.

Finally, achieving equity will require broad advocacy around essential diabetes care and not just advanced technology. Global and local advocates, including the IDF and patient organizations, emphasize that insulin, test strips, and basic screening must be affordable and included in primary care benefit packages. For example, IDF explicitly calls for “advocating for affordable insulin, essential medicines, and diabetes technologies” as part of universal health coverage efforts (19). Recent policy wins illustrate this approach: the 2025 WHO Model List of Essential Medicines was updated to include rapid-acting insulin analogs (20), and NCD alliances are urging countries to revise national formularies and supply policies accordingly (20). At the community level, NGOs and diabetes networks work with governments to integrate diabetes care into health systems (screening programs, task-sharing with community health workers, bulk procurement of insulin, etc.). These system-level strategies, from global meetings and financing agreements to grassroots campaigns, aim to ensure that both lifesaving basics (like insulin) and new technologies are financed, regulated, and delivered equitably to those who need them the most (19, 20).

The digital revolution provides powerful tools against diabetes; however, if left unchecked, it risks creating a two-tier system of “haves” and “have-nots.” Recent studies provide clear evidence that equitable access to technology is a global priority (3). Health systems must scale innovations with equity at the center, not merely for those who can afford them. By forging inclusive partnerships, adopting global standards, and innovating for all contexts, we can ensure that the next generation of diabetes care, from AI analytics to mobile coaching, truly benefits every person with diabetes everywhere. Equity in diabetes technology access must be treated as a global health policy priority and integrated into national strategies and international development agendas.
